# Editorial: Selenium, Human Health and Chronic Disease

**DOI:** 10.3389/fnut.2021.827759

**Published:** 2022-01-18

**Authors:** Barbara R. Cardoso, Cristiane Cominetti, Lucia A. Seale

**Affiliations:** ^1^Department of Nutrition, Dietetics and Food, Monash University, Melbourne, VIC, Australia; ^2^Nutritional Genomics Research Group, Graduate Program in Nutrition and Health, School of Nutrition, Federal University of Goias, Goiania, Brazil; ^3^Pacific Biosciences Research Center, School of Ocean and Earth Science and Technology, University of Hawaii, Honolulu, HI, United States

**Keywords:** selenium, selenoproteins, micronutrients, antioxidant, oxidative stress, ferroptosis

Dietary selenium is a critical factor determining the mineral bioavailability for the synthesis of selenocysteine (Sec)-containing proteins, selenoproteins, which play essential roles in pivotal physiological pathways (1). Selenium deficiency has been implicated in a wide range of chronic diseases, such as cancer, Alzheimer's disease, and thyroid dysfunction (2). Nonetheless, some studies investigate the association between selenium biomarkers and chronic diseases, in an attempt to identify the biological effects of both insufficient and excessive selenium status. Furthermore, with the advance of clinical trials in the field, conflicting information has emerged regarding the benefits of high selenium consumption, and recent research has indicated that supplementation to selenium-replete individuals may be associated with negative health outcomes (3–5).

Considering the aforementioned aspects, this Research Topic aimed to collect scientific articles that bring insights into the fundamental biological role of selenium and shed light on the trade-off between the necessary and harmful levels of selenium intake. In this special e-collection, 12 original and review articles report on selenium metabolism and distribution, suggesting that either deficiency or excess selenium levels may lead to a variety of chronic health issues, including type 2 diabetes *mellitus* (T2DM), non-alcoholic fatty liver disease (NAFLD), and depression.

The review by Ferreira et al. explored the association between gut microbiota and selenium status, focusing on how dietary selenium affects gut microbiome. It provides particular insights into how selenium deficiency may jeopardize the human-microbiota symbiotic relationship, with these symbionts becoming potential competitors and priming the microbiota to be more susceptible to the development of diseases such as cancer, thyroid dysfunctions, and cardiovascular disorders. This comprehensive review also brings novel layers of complexity to selenium metabolism according to the various ingested selenocompounds that can shape future research and the interpretation of selenium biology.

The association between selenium and T2DM was explored in three different studies in this e-collection. The study by Santos et al., conducted with young Brazilian adults with Normal-Weight Obesity (NWO) syndrome, demonstrated that individuals with selenium consumption below the Estimated Average Requirement (EAR; ≤ 45 μg/day) had higher concentrations and a higher prevalence of disturbances in glycated hemoglobin (HbA_1c_) when compared to those with selenium intake above the EAR. In addition, dietary selenium intake was inversely associated with HbA_1c_ concentrations. Contrastingly, Dias et al. observed no significant association between selenium intake and the prevalence of T2DM in a sample of highly educated Brazilian adults. Although these two studies were conducted in the same country, a significant difference was noted in the reported selenium intakes: while in the study of Santos et al. the average intake was below 60 μg/day, the participants in the study by Dias et al. presented a median energy-adjusted selenium intake of 143.5 μg/day, which illustrate geographical and possibly social disparities in the dietary selenium intake. The study by Cardoso et al. investigated the association between blood selenium concentration and glucose markers in US selenium-replete adults. As the main findings, the authors reported a positive association between selenium status, insulin, and the Homeostatic Model Assessment for Insulin Resistance. These studies bring insight into the hypothesis of a U-shaped association between selenium status/exposure and T2DM and corroborate the idea of not promoting supplementation strategies amongst selenium-replete populations.

A U-shaped dose-response in selenium metabolic effects was also raised by Zhang et al. when investigating the association between dietary selenium and incident fracture in Chinese adults. In this 20-year longitudinal study, an increased risk of fracture was found at high selenium intakes as well as at intakes below 30 μg/day. The findings from this study contribute to the current evidence demonstrating that adequate selenium levels, rather than high, are more favorable for health.

Day et al. used publicly available human global gene expression datasets to assess gene expression levels of known selenoprotein pathways in individuals with a healthy liver in comparison to those with NAFLD, whose major risk factor is insulin resistance. The bioinformatics analysis indicates that the NAFLD liver may present lower selenium levels, and that a gene expression variation associated with the metabolism of selenoproteins and iron progresses along with the risk of NAFLD. The findings from this study open avenues for future research that aim to explore the role of selenoproteins in NAFLD pathogenesis.

Almeida et al. investigated the association between selenium intake and depression in a cross-sectional analysis of Brazilian farmers, a rural population poorly assessed. Using a food composition software tailored to the typical Brazilian diet, the authors calculated selenium intake and correlated with the occurrence of depressive episodes, concluding that the prevalence of depression was lower among farmers with the highest intake of selenium. The results align with previous studies in different populations that also demonstrate the same inverse association, and may substantiate improved public health policies toward selenium supplementation to an underserved population.

Three studies were dedicated to the investigation of selenoprotein P (SELENOP), the main selenium transporter. Isobe et al. focused on the association between alcohol intake, serum selenium, and SELENOP concentrations in participants from a rural Japanese town, and found a positive relationship that was independent of age, sex, concentrations of liver enzymes or occurrence of fatty liver. Intriguingly, dose-dependence of this positive association was observed in men, but not in women. The authors argue that a seafood rich diet may contribute to the establishment of this relationship, especially in men.

Kiyohara et al. evaluated the yet unreported biological significance of the interaction between SELENOP1 and Zn^2+^, by examining changes in brain Zn^2+^ in *Sepp1* knockout animals. Changes in the intracellular hippocampal distribution of Zn^2+^ were found in *Sepp*1^−/−^ mice compared to wildtype mice (WT) and this may have been due to a down-regulation of antioxidant selenoproteins. In addition, an increased phosphorylation of tau protein was found in the hippocampus of *Sepp*1^−/−^ mice, possibly resulting from intracellular changes in Zn^2+^. These observations suggested important roles of SELENOP1 in neuronal function, maintenance of synaptic physiology, and prevention of tau hyperphosphorylation, with possible associations with Alzheimer's disease.

Saito elaborated a comprehensive review focused on the molecular mechanisms and role of SELENOP in selenium metabolism, emphasizing that this selenoprotein is not just a selenium transporter, but has a multifunctional role in the maintenance of cellular selenoproteins and in the regulation of cellular redox homeostasis.

The study by Evenson and Sunde uncovers an intriguing perspective regarding how selenium is metabolized. Using rats fed diets containing 0–5 μg of selenite and pulse-injected with 0.5 μg of radiolabeled selenium as various inorganic and organic selenocompounds, the authors could trace the fate of selenium in the body in a dose- and time-dependent manner. Intriguingly, this study demonstrated that all selenocompounds were metabolized into selenium for selenoprotein incorporation without a particular preference. In addition, a portion of selenium was metabolized into “missing” selenocompounds, possibly selenosugars, particularly at shorter time-points, and the distribution may be linked to selenium toxicity and the development of other disease conditions. Despite the limitation of sample size for each radiolabeled compound, there is a breadth of information that this analysis provides on selenium metabolism for the elaboration of new hypotheses and insights that will strengthen the understanding of how selenium and selenoproteins contribute to diseases and health status.

Sex differences in selenium metabolism were explored in the study by Kremer et al. In previous studies the authors produced knockout mouse strains for both *Scly* and *Selenop* genes, the double knockout (DKO) mice. DKO mice showed significant neurological disorders (6), which although less severe in females than in males, were aggravated by the removal of selenium supplementation during puberty (7). In the current special issue, the authors noted that female DKO mice exhibited a particular metabolic phenotype, with significantly higher total body weight and white adipose tissue deposits when compared to WT mice, which was not observed in male counterparts. As the phenotype was reversed by removing selenium from drinking water shortly before puberty, the authors suggested that restricting access to selenium during this period may prevent excessive body weight gain and gonadal fat deposits. In addition, DKO mice have been proven useful as a model for studying the underlying mechanisms and relationships between selenium and energy homeostasis.

In summary, the studies in this e-collection bring novel perspectives and intriguing results on selenium intake, metabolism, distribution, and association with several chronic disease states that burden the health system. The range of unveiled findings will possibly contribute to exciting new hypotheses about the contribution of selenium to human health.

## Author Contributions

All authors contributed to this editorial article and approved the submitted version.

## Conflict of Interest

The authors declare that the research was conducted in the absence of any commercial or financial relationships that could be construed as a potential conflict of interest.

## Publisher's Note

All claims expressed in this article are solely those of the authors and do not necessarily represent those of their affiliated organizations, or those of the publisher, the editors and the reviewers. Any product that may be evaluated in this article, or claim that may be made by its manufacturer, is not guaranteed or endorsed by the publisher.
